# Anti-TNF-α agents Modulate SARS-CoV-2 Receptors and Increase the Risk of Infection Through Notch-1 Signaling

**DOI:** 10.3389/fimmu.2021.641295

**Published:** 2021-05-06

**Authors:** Esra’a Keewan, Shazia Beg, Saleh A. Naser

**Affiliations:** ^1^ Division of Molecular Microbiology, Burnett School of Biomedical Sciences, College of Medicine, University of Central Florida, Orlando, FL, United States; ^2^ UCF Health, College of Medicine, University of Central Florida, Orlando, FL, United States

**Keywords:** COVID19, SARS-CoV-2, TACE, ACE2, Notch, anti-TNF-α, immunity, autoimmune disease

## Abstract

Although millions of patients with underlining conditions are treated primarily with anti-TNF-α agents, little is known about the safety of this standard therapy during the coronavirus disease-2019 (COVID-19) pandemic. In this study, we investigated the effect of anti-TNF-α monoclonal antibodies on the cellular entry mechanism of severe acute respiratory syndrome coronavirus 2 (SARS-CoV-2) and increasing the risk of COVID-19 development. We focused on the expression of angiotensin-converting enzyme II (ACE2), type II transmembrane serine proteases (TMPRSS2)/TNF-α converting enzyme (TACE) ratio. We also investigated the involvement of Notch-1 signaling and its downstream influence on IL-6, myeloid cell leukemia sequence-1(MCL-1) in the anti-TNF-α mode of action and increased the susceptibility to *Mycobacterium avium subspecies paratuberculosis* (MAP) infection. Surprisingly, anti-TNF-α downregulated ACE2 expression by 0.46-fold and increased TMPRSS2/TACE ratio by 44% in THP-1 macrophages. Treatment of macrophages with rIL-6 also downregulated ACE2 and increased TMPRSS2/TACE ratio by 54%. Interestingly, anti-TNF-α treatment upregulated Notch-1, IL-6, and MCL-1 by 1.3, 1.2, and 1.9-fold, respectively, and increased viability and burden of MAP infection in macrophages. Blocking Notch signaling doubled ACE2 expression, decreased TMPRSS2/TACE ratio by 38%, and reduced MAP viability by 56%. In a small group of patients, ACE2 level was significantly lower in the plasma from rheumatoid arthritis (RA) patients on anti-TNF-α treatment compared to healthy control. The data in this critical study demonstrated that through Notch-1/IL-6 signaling, anti-TNF-α agents decreased ACE2 expression and shedding through TMPRSS2/TACE modulation and increased the susceptibility to infection. Overall, this study warns against anti-TNF-α therapy in some patients with underlining inflammatory conditions during the COVID-19 pandemic. The findings should impact current guidelines regarding treatment decisions of patients on anti-TNF-α during the COVID-19 pandemic.

## Introduction

Coronavirus disease 2019 (COVID-19) has emerged as a new respiratory disease caused by severe acute respiratory syndrome coronavirus 2 (SARS-CoV-2). It has been associated with substantial morbidity and mortality, especially in patients with underlining conditions, including autoimmune diseases. Autoimmune disease is a group of conditions that are a broad range of related disorders characterized by the dysregulated immune response, which results in excessive activation of immune cells and subsequent uncontrolled inflammation and tissue damage (1). Overexpression of master pro-inflammatory cytokine tumor necrosis factor alpha (TNF-α) plays a crucial role in the pathogenesis of autoimmune disorders such as rheumatoid arthritis (RA), psoriasis (PsO), ankylosing spondylitis (AS), ulcerative colitis (UC), and crohn’s disease (CD) (2).

Targeting TNF-α by monoclonal antibodies (anti-TNF-α) such as infliximab (IFX), adalimumab (ADA), and certolizumab pegol (CZP) have become the standard treatment of autoimmune diseases (2). However, anti-TNF-α therapy has been ineffective in more than 40% of patients and is associated with adverse effects such as the increased risk of infection, mainly of *Mycobacterium tuberculosis* (MTB) (3–5). More recently, we published a meta-analysis study where a high incidence of MTB infection was reported among CD patients receiving anti-TNF-α agents (odds ratio 5.85) (4). Our laboratory has also shown that anti-TNF-α agents induce *Mycobacterium avium subspecies paratuberculosis* (MAP) survival in infected macrophages (6). MAP is an obligate intracellular pathogen that has been associated with many autoimmune disorders, including RA and CD (7, 8). These findings may explain the poor response of many patients to anti-TNF-α therapy.

Emerging evidence indicates that anti-TNF-α drugs interact with transmembrane TNF-α and elicit reverse signaling cascade in activated macrophages. A process that could modulate TNF-α converting enzyme (TACE) activity (9). TACE (ADAM17) is a member of the ADAM disintegrin and metalloproteinase family, involved in Notch receptor processing (10), which suggests a possible involvement of Notch signaling in the anti-TNF-α mode of action. Notch signaling is a highly conserved juxtacrine signaling, which has been identified as a critical regulator of immune cell development and function (11). Recently, we reported the involvement of Notch-1 signaling in the macrophage response and defense mechanisms against MAP infection (12). Specifically, we unmasked the mechanism involved during MAP infection where MAP survives and causes chronic persistence in macrophages through the induction of Notch-1 signaling and downstream effect on interleukin (IL)-6 and myeloid cell leukemia sequence‐1 (MCL)-1, which ultimately lead to delay in macrophage apoptosis, and subsequent inflammation (12).

TACE has been identified as a principal protease involved in the shedding of angiotensin-converting enzyme II (ACE2), which mediates the entry of SARS-CoV-2 (13). In contrast to ACE1, which catalyzes angiotensin II formation, ACE2 antagonizes ACE1 activity by converting angiotensin II to angiotensin 1-7 (14). The ACE2/Angiotensin 1-7 axis has anti-inflammatory, antioxidant, anti-fibrosis, anti-apoptosis, and cardiovascular protection effects (15). Recombinant ACE2 has been proposed as a potential therapeutic option for SARS-CoV-2 infection and complications (15). ACE2 cleavage by TACE, resulting in the release of the ACE2 ectodomain into the circulation (13). Suggests a possible TACE involvement in controlling SARS-CoV-2 infection through enhancing ACE2 shedding and increasing free catalytic active ACE2 in the circulation to neutralize SARS-CoV-2 and prevent its interaction with other receptors. Most importantly, this may block the spread of SARS-CoV-2 and protect from developing acute lung failure and respiratory distress syndrome by regulating the renin-angiotensin system. It has been proposed that ACE1/ACE2 imbalance exaggerates renin‐angiotensin signaling leading to lung injury in COVID-19 (14). Specifically, it has been proposed that SARS-CoV-2 infection causes inhibition in catalytic activity and possible expression of ACE2 (16). If true, this would be detrimental in the elderly population and those with underlining conditions who already have lower ACE2 levels compared to younger and healthy groups (17). This becomes even more concerning in RA and inflammatory bowel disease (IBD) patients who are on anti-TNF-α therapy despite the fact that it is not clear how anti- TNF-α therapy may dysregulate ACE2 expression and shedding. We became intrigued by this observation, especially after the recent COVID-19-advisory from the Crohn’s and Colitis Foundation of America (CCFA) to IBD patients to pause or stop anti-TNF-α therapy if exposed or infected with SARS-CoV-2 (18). The type II transmembrane serine proteases (TMPRSS2) have also been reported to be essential for viral entry to the host cells (19, 20). Specifically, TMPRSS2 mediated S1/S2 subunits proteolytic cleavage upon binding with ACE2, facilitating the virus entrance to the host cells (19). Interestingly, TMPRSS2 was reported to interfere with TACE activity and inhibit ACE2 shedding (20).

Although millions of patients with underlining conditions, including RA and CD, are treated primarily with anti-TNF-α agents, little is known about the safety of this standard therapy and susceptibility to SARS-CoV-2 infection during COVID-19 pandemic. In this study, we investigated the effect of anti-TNF-α monoclonal antibody therapy on the cellular entry mechanism of SARS-CoV-2 and the risk of COVID-19 development. We focused on the expression and dysregulation of ACE2, TMPRSS2, and TACE. We also investigated the mechanism involved in Notch-1 signaling and downstream influence on IL-6, MCL-1, and intracellular infection by MAP in macrophages treated with anti-TNF-α drugs. We also investigated the ACE2 level in plasma from RA patients on anti-TNF-α therapy and controls.

## Materials and Methods

### Cell Culture and Treatment

The human monocytic cell line THP-1 (ATCC TIB-202) was used to model macrophages. THP-1 cells were cultured in RPMI-1640 medium (Thermo Fisher, Cat# A1049101, Waltham, MA, USA) supplemented with 10% fetal bovine serum (FBS) (Thermo Fisher, Waltham, MA, USA) and kept in a humidified 5% CO₂ incubator at 37°C. THP-1 monocytes were differentiated to THP-1 derived macrophages by 50 ng/ml of phorbol 12-myristate 13-acetate (PMA) (Sigma-Aldrich, Missouri, USA) for 48 hours.

Notch signaling in THP-1 derived macrophages was targeted pharmacologically using γ-secretase inhibitor DAPT [N‐(N‐[3,5‐difluorophenacetyl] ‐l‐alanyl) ‐S‐phenylglycine t‐butyl ester] (CST, Danvers, MA, USA). To examine the effect of anti-TNF-α therapy on Notch signaling and its downstream effect, THP-1 derived macrophages were treated with two forms of anti-TNF-α agents, non-PEGylated (ADA), and PEGylated (CZP).

### Bacterial Culture

Clinical MAP strain (UCF4; isolated from CD patient) was cultured in Bactec MGIT Para-TB medium tubes (Becton Dickinson, NJ, USA), containing Bactec MGIT Para-TB supplements (Bovine albumin, Catalase, Casein, and Oleic acid) and incubated in BD BactecTM MGITTM 320 Analyzer at 37°C.

### Quantitative Real-Time PCR (RT-PCR)

TRIzol™ reagent was used for total RNA isolation from cells (Thermo Fisher, Waltham, MA, USA) according to the manufacturer’s instructions. Briefly, 800 ng of total RNA was used to synthesize cDNA, and quantitative real-time PCR was performed using the StepOnePlus™ Real-Time PCR System (Thermo Fisher, Waltham, MA, USA) with Fast SYBR Green Mastermix (Thermo Fisher, Waltham, MA, USA) as detection dye. A housekeeping β-actin primer (Thermo Fisher, Waltham, MA, USA) was used to measure the endogenous baseline CT values. Relative mRNA expression levels were calculated by using the equation 2^(−ΔCT)^ *1000, where ΔCT= [(Sample RT-PCR CT value) − (β-actin CT baseline value)]. The primers (Thermo Fisher) used for the RT-PCR in this study are shown in **Table 1**.

**Table 1:** Primer sequences of genes used in this study.

**Gene**	**Primer Sequence (5’-3’)**	**Amplicon Length (bp)**
**β-actin**	CTCATCTTGTTTTCTGCGCAAGTT CTTCCCTCCTCAGATCATTGCTC	226
**Notch-1**	TGAAATTCAGGGCCCCTCC GCATCGGGCACCTGAAC	162
**IL-6**	AGGAGAAGATTCCAAAGATGTAGCC TGCTCTAGAACCCAGCAAAGAC	228
**MCL-1**	TGGTGGTGGTTGGTTAAAAGTCA GTGGAGTTCTTCCATGTAGAGGAC	152
**ACE2**	CTCCCTCTCAGGCATAACTTGG TGAGTTCACGATAGAAAAATTGAAGGAAGA	262
**TACE**	CTCGTCCATATGTGAGTCTGTGC TTATTCCTATCAATCAATTGAACCCATGTCTT	226
**TMPRSS2**	AATAAAAATGAAGTGACCTCTGAATCATCTCT AAGCTTGCTTATTTGGTTTCTAAGTGC	238

### Measurement of MAP Viability

Live/Dead™ Baclight™ bacterial viability assay (Thermo Fisher, Waltham, MA, USA) was used to measure relative MAP viability in infected THP-1 derived macrophages. Briefly, macrophages were infected with MAP (UCF 4, 10^7^ CFU/ml) for 24 hours. Cells were then washed with phosphate buffer saline (PBS) (Thermo Fisher, Waltham, MA, USA) and then collected and lysed with 500 µL of M-PER Mammalian reagent (Thermo Fisher, Waltham, MA, USA). After sample centrifugation, 100 μL of each sample supernatant was loaded in triplicates in 96-well microplate and mixed with 100 μL of staining reagent mixture (SYTO^®^ 9 green- and Propidium iodide red-fluorescent nucleic acid stains). Following 15 minutes of incubation in the dark, the integrated intensities of the green (530 nm) and red (630 nm) emission and excitation at 485 nm were measured using SpectraMAX^®^ i3x Multi-mode microplate reader. Then the green/red fluorescence ratios were calculated, which detected the Live/Dead MAP ratio.

### Clinical Samples

Peripheral blood (4.0-ml K2-EDTA coded tube) samples were obtained from 21 subjects (9 RA patients on anti-TNF-α and 12 healthy controls). All participants completed and signed a written informed consent prior to enrollment in the IRB-approved study #IRB00001138. Plasma was isolated and maintained at -20C˚ for later use.

Measurement of Circulating ACE2 Levels by Enzyme-Linked Immunosorbent Assay (ELISA).

Circulating ACE2 levels were measured in the plasma using Soluble Angiotensin-Converting Enzyme 2 (ACE2) (Human) (AB, CA, USA) following the manufacturer’s instructions. ACE2 levels were determined by reading optical density at 450 nm using SpectraMAX^®^ i3x Multi-mode microplate reader.

### Statistical Analysis

To determine the statistical significance in this study, all data were pre-tested for normal distribution using the Kolmogorov–Smirnov normality test followed by paired tow-tailed t-test to determine the difference between the treated groups and controls, and unpaired tow-tailed t-test to determine the difference in the plasma ACE2 levels between RA patients and healthy controls. All experiments were performed in triplets, and values are presented as mean ± standard deviation (SD). All statistical analyses were performed using Prism GraphPad software (version 8), with P<0.05 considered significant.

## Results

### Anti-TNF-α Agents Induce Notch-1, IL-6 and MCL-1 Expression in Macrophages

**Figure 1:**
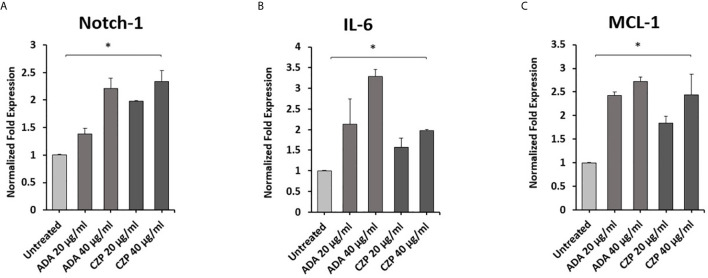
Anti-TNF-α agents induce Notch-1 and its downstream effect on IL-6 and MCL-1. THP-1 derived macrophages were treated with non-PEGylated (ADA) and PEGylated (CZP) anti-TNF-α agents (0-40 μg/ml). The expression level of **(A)** Notch-1, **(B)** IL-6, and **(C)** MCL-1 were detected by RT-PCR. All experiments were performed in triplets. Data are shown as mean ± SD, and significance is considered as *P < 0.05.

To explore the effects of therapeutics approved anti-TNF-α agents on Notch-1 and its downstream effect on IL-6 and MCL-1 expression in macrophages. THP-1 derived macrophages were treated with two forms of anti-TNF- α agents, non-PEGylated (ADA, 0-40 μg/ml), and PEGylated (CZP, 0-40 μg/ml). We found that anti-TNF-α agents induce the expression of Notch-1, IL-6, and MCL-1 in THP-1 derived macrophages in a dose-dependent manner, at 40 μg/ml concentration level, non-PEGylated (ADA): 1.2, 2.2, and 1.7-fold and PEGylated (CZP): 1.3, 1.0 and 1.4-fold, respectively) compared with untreated groups (P < 0.05) (**Figures 1A–C**).

### Effect of Anti-TNF-α on Notch-1, IL-6 and MCL-1 Expression in MAP Infected Macrophages

**Figure 2:**
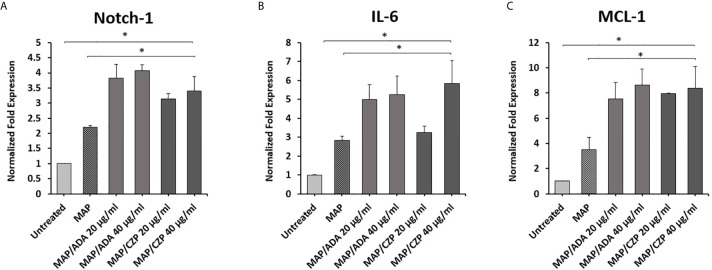
Anti-TNF-α agents potentiate Notch-1 and its downstream effect on IL-6 and MCL-1 expression in MAP infected macrophages. MAP infected-THP-1 derived macrophages were treated with non-PEGylated (ADA) and PEGylated (CZP) anti-TNF-α agents (0-40 μg/ml). The expression level of **(A)** Notch-1, **(B)** IL-6, and **(C)** MCL-1 were detected by RT-PCR. All experiments were performed in triplets. Data are shown as mean ± SD, and significance is considered as *P < 0.05.

To determine whether anti-TNF-α agents potentiate MAP impact in infected macrophages. THP-1 derived macrophages were infected with MAP (10^7 CFU/ml) for 24 hours. Then the infected macrophages were treated with two forms of anti-TNF-α agents, non-PEGylated (ADA)(0-40 μg/ml) and PEGylated (CZP) (0-40 μg/ml). As shown in **Figure 2**, MAP infection significantly induced the expression of Notch-1, IL-6, and MCL-1 by 1.3, 1.2, and 1.9-fold, respectively, compared to untreated groups. Also, Anti-TNF-α agents significantly induced Notch-1, IL-6, and MCL-1 expression in infected macrophages, at 40 μg/ml concentration level, non-PEGylated (ADA): 1.8, 2.4, and 5. 0-fold and PEGylated (CZP): 1.2, 3.0, and 5. 0-fold, respectively, compared with MAP infected (untreated) macrophages (P < 0.05) (**Figures 2A–C**).

### Effects of Anti-TNFα/Notch-1 on Modulating MAP Viability in Infected Macrophages

**Figure 3:**
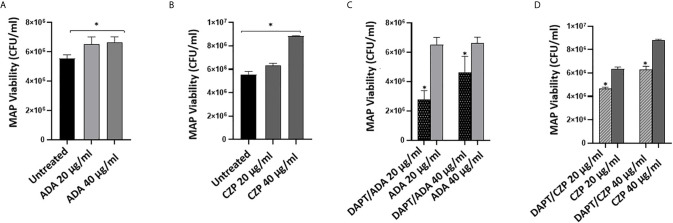
DAPT prevents anti-TNF-α induces MAP survival in macrophages. **(A, B)** MAP viability in non-PEGylated (ADA) (0-40 μg/ml) and PEGylated (CZP) (0-40 μg/ml) anti-TNF-α treated macrophages. **(C, D)** MAP viability in pre-infection DAPT (30 μM)/post-infection PEGylated (0-40 μg/ml) and PEGylated (0-40 μg/ml) anti-TNF-α treatment. MAP viability was measured using Live/Dead Baclight bacterial viability assay. MAP viability is represented as (CFU/ml). All experiments were performed in triplets. Data are shown as mean ± SD, and significance is considered as *P < 0.05.

We previously reported that anti-TNF-α agents induce MAP survival in infected macrophages. In this study, we have confirmed the involvement of Notch-1 signaling in anti-TNF-α agents’ mode of action (**Figures 1** and **2**). To further confirm the involvement of Notch signaling in anti-TNF-α agents mediated MAP survival in infected macrophages, we examined the effect of anti-TNF-α agents on MAP viability in macrophages pre-treated with DAPT (30 μM/24 hours). As shown in **Figures 3A, B**, anti-TNF-α agents promoted MAP survival in infected macrophages in a dose-dependent manner. However, DAPT pre-treatment dramatically diminished the ability of anti-TNF-α agents to sustain MAP survival in infected macrophages by 26%-56% in the two forms of anti-TNF-α agents treated groups compared with untreated groups (P < 0.05) (**Figures 3C, D**).

### Effect of Anti-TNF-α on ACE2, TACE, and TMPRSS2 Expression in Macrophages

**Figure 4:**
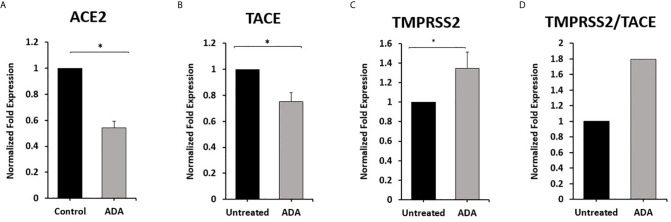
Anti-TNF-α inhibit ACE2 and TACE expression and enhance TMPRSS2 in THP-1 derived macrophages. THP-1 derived macrophages were treated with non-PEGylated anti-TNF-α (ADA; 40 μg/ml). **(A–C)** The expression levels of ACE2, TACE, and TMPRSS2 were detected by RT-PCR. **(D)** The ratio between TMPRSS2 and TACE. All experiments were performed in triplets. Data are shown as mean ± SD, and significance is considered as *P < 0.05.

To explore the involvement of anti-TNF-α therapy in the cellular entry of SARS-CoV-2 and the development of COVID-19. THP-1 derived macrophages were treated with non-PEGylated anti-TNF- α (ADA; 40 μg/ml) for 24 hours. Then we measured the expression of ACE2, TACE, and TMPRSS2. As shown in **Figures 4A, B**, anti-TNF- α significantly inhibited ACE2 and TACE expression in macrophages by 0.46 and 0.25-fold compared with untreated macrophages (P < 0.05). Whereas anti-TNF- α induced the expression of TMPRSS2 by 0.35-fold and increased TMPRSS2/TACE ratio by 44% compared with the untreated groups (P < 0.05) (**Figures 4C, D**).

### Effect of Notch-1/IL-6 on ACE2, TACE, and TMPRSS2 Expression in Macrophages

**Figure 5:**
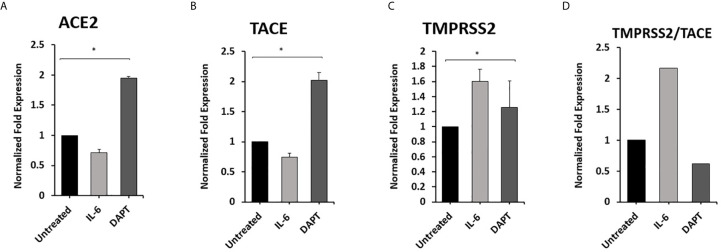
Notch-1 and rIL-6 inhibit ACE2 and TACE expression and enhance TMPRSS2 in THP-1 derived macrophages. THP-1 derived macrophages were treated with DAPT (30 μM) and rIL-6 (500 U/ml) for 24 hours. **(A–C)** The expression levels of ACE2, TACE, and TMPRSS2 were detected by RT-PCR. **(D)** The ratio between TMPRSS2 and TACE. All experiments were performed in triplets. Data are shown as mean ± SD, and significance is considered as *P < 0.05.

To examine the effect of Notch-1 and IL-6 on ACE2, TACE, and TMPRSS2 Expression in Macrophages. THP-1 derived macrophages were treated with DAPT (30 μM) and rIL-6 (500 U/ml) for 24 hours. We found that targeting Notch signaling using DAPT induced the expression of ACE2 and TACE by 0.8 and 1.0-fold compared with untreated groups (P < 0.05) (**Figures 5A, B**). DAPT treatment induced TMPRSS2 expression by 0.25-fold and decreased TMPRSS2/TACE ratio by 38% compared with untreated groups (P < 0.05) (**Figures 5C, D**). rIL-6 treatment decreased ACE2 and TACE expression by 0.3 and 0. 26-fold, respectively, compared with untreated groups (P < 0.05) (**Figures 5A, B**). rIL-6 increased TMPRSS2 expression by 0.6-fold and TMPRSS2/TACE ratio by 54% compared with untreated groups (P < 0.05) (**Figures 5C, D**).

### Anti-TNF-α and rIL-6 Modulate ACE2, TACE and TMPRSS2 Expression in Macrophages via Notch Signaling

**Figure 6:**
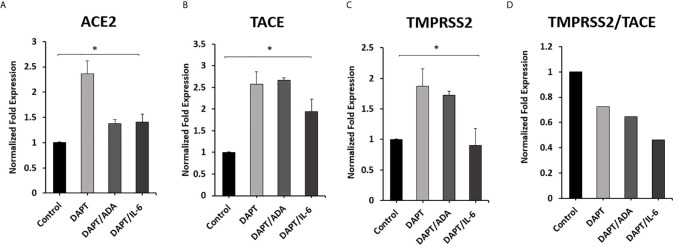
Effect of anti-TNF-α and rIL-6 on ACE2, TACE, and TMPRSS2 Expression in DAPT pretreated Macrophages. THP-1 derived macrophages were pre-treated with DAPT (30 μM for 24 h) and then treated with non-PEGylated anti-TNF-α (ADA; 40 μg/ml) and rIL-6 (500 U/ml). The expression levels of **(A)** ACE2, **(B)** TACE, and **(C)** TMPRSS2 were detected by RT-PCR. **(D)** The ratio between TMPRSS2 and TACE. All experiments were performed in triplets. Data are shown as mean ± SD, and significance is considered as *P < 0.05.

To investigate the involvement of Notch signaling in anti-TNF-α and rIL-6 mediated effect on ACE2, TACE, and TMPRSS2 Expression in macrophages. The impact of anti-TNF-α and rIL-6 on ACE2, TACE, and TMPRSS2 Expression in macrophages pre-treated with DAPT (30 μM for 24 h) was examined. As shown in **Figures 6A, B**, DAPT pretreatment significantly diminished the ability of anti-TNF-α and rIL-6 to decrease the expression of ACE2 and TACE and induced the expression of ACE2 and TACE in anti-TNF-α treated groups (0.4 and 1.7-fold, respectively) and in rIL-6 treated groups (0.4 and 0.9-fold, respectively) compared with untreated groups (P < 0.05). Even DAPT pretreatment doesn’t significantly modulate TMPRSS2 expression in the presence of anti-TNF-α and rIL-6 (**Figure 6C**). However, DAPT pretreatment significantly decreased TMPRSS2/TACE ratio in anti-TNF-α treated groups by 35% and in rIL-6 treated groups by 54% compared with untreated groups (P < 0.05) (**Figure 6D**).

### Circulating Plasma ACE2 levels in RA Patients on Anti-TNF-α Therapy

**Figure 7:**
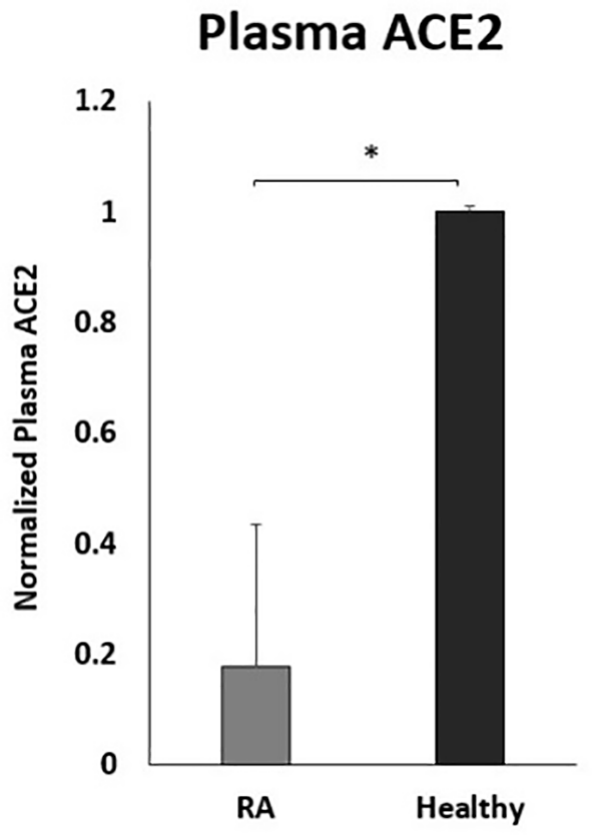
Decreased plasma ACE2 levels in RA patients on anti- TNF-α treatments. RA patients (n=9) and healthy control (n=12). ACE2 levels were detected by ELISA. Data are shown as mean ± SD, and significance is considered as *P < 0.05.

To examine the effect of anti-TNF-α on ACE2 shedding, the circulating ACE2 levels were examined in the plasma of a small number of RA patients on anti-TNF-α therapy. As shown in **Figure 7**, the ACE2 plasma levels were significantly lower in RA patients on anti-TNF-α therapy compared with healthy control.

## Discussion

Notch signaling is juxtacrine signaling regulating critical cellular processes like proliferation, differentiation, and apoptosis (11). Notch signaling dysregulation was associated with many pathological settings, including autoimmune diseases (11, 21, 22). However, so far there is no report about the effect of autoimmune medications on Notch signaling. Recently we reported the involvement of Notch signaling and its downstream impact on IL-6 and MCL-1 in macrophages immune response and defense mechanisms against MAP infection (12), the bacteria that have been associated with many autoimmune diseases like RA and CD (7, 8). Additionally, we reported that the anti-TNF-α agents, which are considered the cornerstone in treating autoimmune diseases, increased MAP viability in MAP infected macrophages (6). Intriguingly, A reverse signaling mechanism of anti-TNF-α agents could modulate TACE which in turn modulates Notch-1 cleavage and its subsequent activation (9, 10). All this evidence prompted us to examine the potential involvement of Notch signaling in the anti-TNF-α mode of action.

In this study, we were able to confirm the involvement of Notch-1 signaling in the anti-TNF-α mode of action. ADA and CZP significantly induced the expression of Notch-1 in macrophages. Furthermore, ADA and CZP treatment induced the expression of IL-6 and MCL-1 in macrophages, a finding that could explain the poor response of anti-TNF-α agents in a substantial number of patients. Many studies supported the reciprocal modulation between Notch signaling and various pro-inflammatory cytokines such as TNF-α and IL-6 in macrophages, ultimately resulting in the sustained inflammatory response in macrophages (11). Accordingly, anti-TNF-α ability to induce the expression of Notch-1 and IL-6 could start a feed-forward loop to fuel the immune response of macrophages. Additionally, targeting MCL-1, the antiapoptotic protein, shows an anti-inflammatory effect by enhancing bacterial clearance and accelerate inflammatory resolution in sterilizing and infected macrophages (23). Recently, we reported the reciprocal modulation between Notch-1 and IL-6 signaling to hijack the MCL-1 dependent inhibition of apoptosis and increase bacterial load and persistence and successive inflammation (12).

Thus, we are intrigued to study the behavior of anti-TNF-α agents in the presence of MAP infection. We found that ADA and CZP significantly potentiate MAP mediated effect on macrophages by amplifying the expression of Notch-1, IL-6, and MCL-1 in MAP infected macrophages. Thereafter, we hypothesized that anti-TNF-α modulates macrophages defense mechanisms against intracellular pathogens such as MAP through Notch-1 signaling. Thus, we targeted Notch signaling using DAPT, and we found targeting Notch signaling prevents anti-TNF-α induces MAP survival in infected macrophages. This novel finding of the involvement of Notch-1 signaling in the anti-TNF-α mode of action could potentially explain the poor response to these agents, particularly in MAP positive individuals.

The association of anti-TNF-α agents and intracellular infection prompted us to extend our effort to examine these agents safety during COVID-19 pandemic. COVID-19 has emerged as a new respiratory disease caused by SARS-CoV-2. COVID-19 has been associated with substantial morbidity and mortality, especially in patients with underlining conditions (24). Millions of patients with underlining conditions, including those with CD and RA, are treated primarily with anti-TNF-α monoclonal antibodies. A recent alarming advisory from CCFA and other national organizations advised patients on anti-TNF-α therapy to pause or stop taking the medications if exposed or became infected with SARS-CoV-2 (18). Still, there are compelling arguments about the safety of these medications during COVID-19 infection. For instance, some recent reports suggested anti-TNF-α agents as a potential therapy for COVID-19 (25). However, Little is known about the mechanistic effect of these medications during SARS-CoV-2 infection.

In this study, we investigated *in vitro* the potential involvement of anti-TNF-α therapy in the cellular entry of SARS-CoV-2 and the development of COVID-19. Specifically, we determined the interaction of anti-TNF-α with TACE and subsequent modulation of SARS-CoV-2 cell receptor ACE2. ACE2 plays a critical role in regulating the renin-angiotensin-aldosterone system, which subsequently regulates blood pressure and fluid balance (17), and also shows anti-inflammatory effects and protects organs from inflammatory injuries (15). ACE2 is subjected to cleavage by two membrane proteases TACE and TMPRSS2. TMPRSS2-cleaved ACE2 facilitates SARS-CoV-2 cellular entry, whereas TACE-cleaved ACE2 prevent virus entry and provide protection to the organs (20). The SARS-CoV-2 infection resulted in ACE2 dysfunction and worsened COVID-19 by dysregulating the renin-angiotensin-aldosterone system, leading to multiorgan failure (26). Thus, in this study, we were interested in measuring TMPRSS2/TACE expression ratio as an indicator of ACE2 shedding. Our data show a decrease in ACE2 and TACE expression in response to ADA treatment, coincidence with increase TMPRSS2/TACE ratio. Interestingly, a study based on lung transcriptome analysis of severe COVID-19 patients showed high ACE2 expression (27), another study reported that pathological alterations in lungs were significantly decreased in ACE2 knockout mice (28). They believed that the negative effect of SARS-CoV infection on the renin-angiotensin system might be mediated by modulating ACE2 activity, which may cause severe acute lung failure (28). This finding suggests that anti-TNF-α potentiate SARS-CoV-2 action and causes dysregulation of ACE2 expression and facilitates the virus entry by enhancing TMPRSS2-cleaved ACE2 versus TACE-cleaved ACE2. Low levels of ACE2 were reported in many autoimmune diseases and elderly individuals, which make those patients more vulnerable to COVID-19 infection and subsequent severe outcomes (16). For example, impaired ACE2 gene expression level was reported in CD patients and associated with worse outcomes in those patients, supported the protective effect of ACE2 in autoimmune diseases (29).

The elevated level of IL-6 was associated with a high case fatality of COVID-19 infection (30). Given the reciprocal modulation of Notch-1 and IL-6, we investigated the effect of IL-6 and Notch signaling on ACE2 expression and cleavage processes. Consistent with anti-TNF-α data, IL-6 also inhibits the expression of ACE2 and TACE and increases ACE2 cleavage in favor of TMPRSS2. This process enhances virus entry to the cells and dysregulates the renin-angiotensin-aldosterone system. On the other hand, targeting Notch signaling using DAPT enhances ACE2 and TACE2 expression and significantly reduces TMPRSS2/TACE ratio. This led us to examine if anti-TNF-α modulate ACE2 expression and processing through Notch signaling. We found that targeting Notch signaling resulted in a loss of the inhibitory effect of anti-TNF-α and IL-6 on ACE2 and TACE, lead to a decrease TMPRSS2/TACE ratio. This finding strongly suggests the potential role of Notch signaling in the virus entry mechanism through its regulatory effects on the ACE2 receptor.

Circulating ACE2 (plasma ACE2) is produced after being shed from cell membranes due to membrane protease effects (31). A high level of circulating ACE2 has been associated with a better outcome in patients with influenza A H7N9 infection (32). On the contrary, ACE2 knockout led to severe lung damage in H7N9 infected mice, while recombinant ACE2 reduced associated lung injury (32). This suggests that circulating ACE2 may competitively bind with SARS-CoV-2 to neutralize the virus and rescue cellular ACE2 activity to protect the lung from damage. Interestingly, reduced plasma ACE2 in the plasma of COVID-19 patients led to reducing the ability to prevent SARS-CoV-2 binding to host cells (33). Our data indicated a remarkable decrease in circulating ACE2 in RA patients on anti-TNF-α treatment compared with healthy control. Raises potential concerns about the safety of these medications during the COVID-19 pandemic.

In conclusion, our study significantly extends the current knowledge of the anti-TNF-α therapy mode of action in patients during the COVID-19 pandemic. This is a rapidly changing time and the information we know daily should help address and control the COVID-19 pandemic. The data clearly associates anti-TNF-α therapy with an increase in the risk of intracellular infection, including MAP and SARS-CoV-2 infection *via* induction of Notch-1 signaling. Specifically, our results show that anti-TNF-α agents increase MAP viability in infected macrophages through the induction of Notch-1 signaling and its downstream effect on IL-6 and MCL-1. Additionally, we have demonstrated a disadvantage for the use of anti-TNF-α therapy due to its effect on ACE2 expression and shedding mechanism through Notch signaling, a process that favors SARS-CoV-2 cellular entry. Consequently, this exaggerates the viral infection, and significantly increases cytokine production (cytokine storm). Overall, this innovative study demonstrates that anti-TNF-α therapy is a high-risk factor in patients with underlining inflammatory conditions during the COVID-19 pandemic. The outcome of this study will impact current guidelines regarding treatment decisions of patients on anti-TNF-α during the COVID-19 pandemic. Additionally, it provides a powerful and promising strategy (targeting Notch signaling) to combat infection and inflammation during SARS-CoV-2 infection. A large-scale study should be performed to look at the level of ACE2 in a large number of blood samples from patients on various treatment options in order to establish a true conclusion about the effect of anti-TNF-α on ACE2 level in patients

## Data Availability Statement

The raw data supporting the conclusions of this article will be made available by the authors, without undue reservation.

## Ethics Statement

The studies involving human participants were reviewed and approved by University of central Florida IRB (#IRB00001138). The patients/participants provided their written informed consent to participate in this study.

## Author Contributions

EK designed and performed experiments, analyzed the data, and prepared the manuscript. SB has kindly supervised and provided the clinical samples used in this study and has participated in all aspects of the study. SN is the mentor and primary advisor who supervised all aspects of the work including designing the experiments, interpreting the data, writing and editing the manuscript. All authors contributed to the article and approved the submitted version.

## Funding

This work was funded, in part, by the Florida Legislative Grant and the UCF College of Medicine Dean’s Research.

## Conflict of Interest

The authors declare that the research was conducted in the absence of any commercial or financial relationships that could be construed as a potential conflict of interest.
